# Expression and regulation of HIF-1alpha in macrophages under inflammatory conditions; significant reduction of VEGF by CaMKII inhibitor

**DOI:** 10.1186/1471-2474-11-61

**Published:** 2010-03-30

**Authors:** Johanna Westra, Elisabeth Brouwer, Ingrid AM van Roosmalen, Berber Doornbos-van der Meer, Miek A van Leeuwen, Marcel D Posthumus, Cees GM Kallenberg

**Affiliations:** 1 Department of Rheumatology and Clinical Immunology, University Medical Center Groningen, Groningen, the Netherlands

## Abstract

**Background:**

Macrophages expressing the pro-angiogenic transcription factor hypoxia-inducible factor (HIF)-1alpha have been demonstrated in rheumatoid arthritis (RA) in the synovial tissue. Aim of the present study was to investigate intracellular signal transduction regulation of pro-inflammatory HIF-1 alpha expression in macrophages to identify possible new intervention strategies. We investigated the effects of CaMKII-inhibitors amongst other kinase inhibitors, on HIF-1 alpha expression and downstream production of pro-angiogenic factors in macrophages.

**Methods:**

Differentiated THP-1 cells and synovial fluid (SF) macrophages were stimulated with 1 μg/ml LPS with or without pretreatment with specific inhibitors of the ERK pathway (PD98059), the PI3K pathway (LY294002), and the CaMKII pathway (KN93 and SMP-114). mRNA and protein expression of HIF-1 alpha, VEGF, MMP-9, and IL-8 was measured in cell lysates and cell supernatants.

**Results:**

HIF-1 alpha protein expression in LPS-stimulated THP-1 macrophages could be blocked by ERK- and PI3K-inhibitors, but also by the CaMKII inhibitor KN93. THP-1 and SF macrophages produced high levels of VEGF, IL-8, and MMP-9, and VEGF protein production was significantly inhibited by PI3K-inhibitor, and by both CaMKII inhibitors. LPS stimulation in an hypoxic environment did not change VEGF levels, suggesting that LPS induced VEGF production in macrophages is more important than the hypoxic induction.

**Conclusions:**

Expression of HIF-1 alpha and downstream effects in macrophages are regulated by ERK-, PI3K, but also by CaMKII pathways. Inhibition of HIF-1α protein expression and significant inhibition of VEGF production in macrophages was found using CaMKII inhibitors. This is an unknown but very interesting effect of the CaMKII inhibitor SMP-114, which has been in clinical trial as DMARD for the treatment of RA. This effect may contribute to the anti-arthritic effects of SMP-114.

## Background

Macrophages are known to play an important role in inflammatory diseases such as rheumatoid arthritis (RA), as the rheumatoid synovium is intensively infiltrated by macrophages and their numbers correlate well with articular destruction [1] and clinical scores [2]. It has long been recognized that synovial fluids from RA patients are hypoxic, acidotic and have low glucose and high lactate levels [3]. This is indicative of an anaerobe situation, which has been confirmed by measuring oxygen levels in the synovium. [4]. A microenvironment of hypoxia leads to the formation of an ubiquitously expressed transcription factor, hypoxia-inducible factor (HIF-1), which regulates the expression of genes that allows cells to use anaerobic metabolism to generate energy for survival and secondly, to promote angiogenesis for oxygen supply [5]. The heterodimeric transcription factor HIF is composed of two basic helix-loop-helix (bHLH) proteins (HIF-1α and HIF-1β). The HIFα/β dimer binds to a core DNA motif in the hypoxia responsive elements, which are associated with a broad range of target genes, such as vascular endothelial growth factor (VEGF), erythropoietin (EPO), and glucose-transporter-1 (GLUT-1), promoting angiogenesis, erythropoiesis, cell growth and migration, and a switch to a glytolytic cell metabolism [6]. HIF-1β, also known as ARNT (aryl hydrocarbon receptor nuclear transporter) is constitutively expressed, whereas HIF-1α is induced, amongst other stimuli, by hypoxia. During normoxia HIF-1α is hydroxylated at specific prolyl residues leading to degradation through the ubiquitin-proteasome pathway [7,8]. However, under normoxic circumstances HIF-1α can be stabilized in cell lines and primary cell-cultures by other stimuli, such as mechanical stress, hormones, cytokines, growth factors but also by reactive oxygen and nitrogen particles [9]. In ligand-induced activation of HIF-1, in general two major phosphorylation pathways are involved, the phosphatidylinositol-3-kinase (PI3K) and the mitogen-activated protein kinase (MAPK) pathway [10]. Frede *et al *[11] reported involvement of the ERK (p44/42) MAPK pathway in differentiation of the human monocytic cell line THP-1 along with increased HIF-1 activity, while increased expression of HIF-1α correlated to differentiation was also reported by others [12].

In recent reviews the possible important role of HIF-1 in RA is extensively discussed [6,13]. Especially the presence of both hypoxia and inflammatory proteins in RA both leading to HIF-1α stabilization and subsequent HIF-1 activation seems to warrant an important role for HIF-1α. Recently new small molecular drugs that have inhibitory effect on HIF-1α have been tested in arthritis models. Effects of 2 ME-2 (methoxyestradiol) were investigated in a rat CIA model and in a rat AIA model [14,15]. In the CIA model a marked suppression of synovial gene expression of bFGF and VEGF was observed, with parallel reduction of synovial blood vessels, whereas in both CIA and AIA the severity of disease was reduced. Inhibitors of Hsp90 have been shown to inhibit HIF-1 activity and were investigated *in vitro *and *in vivo *in arthritis models. They showed to inhibit paw swelling and to improve body weight. Scores for inflammation, pannus formation, cartilage damage, and bone resorption returned to normal [16].

Recently, involvement of another signal transduction pathway in HIF-1 transcriptional activity was reported, namely the Ca^2+^/Calmodulin-dependent kinase II (CaMKII) pathway [17]. Many of the cellular responses to Ca^2+^are modulated by a family of protein kinases, namely Ca^2+^/calmodulin dependent protein kinases (CaMK), among which CaMKII is ubiquitously expressed. CaMKII has been reported to play an important role in osteoclast differentiation and function [18] and to be expressed in macrophages and fibroblasts in RA synovial tissue, and also in cultured synovial fibroblasts (Tagashira S *et al*, postersession A, no 94, ACR meeting, Washington 2006). Recently it was shown that CaMKII activation was involved in TLR-triggered, pro-inflammatory cytokine production by macrophages [19].

In this study we investigated expression of HIF-1α in macrophages with subsequent activation both in an inflammatory and hypoxic environment, and evaluated whether this activation leads to production of proangiogenic factors. Moreover we studied the effect of specific signal transduction inhibitors both on HIF-1α expression and on downstream products of HIF-1 activation in macrophages in cell-lines as well as in macrophages isolated from synovial fluid (SF). We, hereby, included the use of a novel CaMKII inhibitor, which has been shown to have excellent efficacy in collagen-induced arthritis in rats (Furuichi *et al*., abstract Fri0027, EULAR, Barcelona 2007) and which has been in phase IIb clinical trial in Europe.

## Methods

All chemicals used were from Sigma Chemical Co., St. Louis, MO, unless otherwise indicated. RPMI 1640 medium and gentamycin were purchased from Gibco (Life Technologies Ltd, Paisley, Scotland). Fetal calf serum (FCS) was from BioWhittaker Europe (Verviers, Belgium), and culture plates from Costar (Badhoevedorp, The Netherlands). NE-PER^® ^Nuclear and Cytoplasmic Extraction Reagents were obtained from Pierce Technology (Rockford, IL). Anti- HIF-1α for Western Blotting was from BD Transduction labs. (BD Biosciences, Breda, the Netherlands); anti-HIF-1alpha 67sup (nr 463) for immunohistochemistry was from Abcam (Cambridge, UK). The signal transduction inhibitors LY294002, PD98059, KN-93, and the HIF-1α inhibitor YC-1 were purchased from Calbiochem (via Merck Eurolab, Amsterdam, The Netherlands). SMP-114 was supplied by Dainippon Sumitomo Pharma (Osaka, Japan). All reagents for RNA isolation and reverse transcriptase reaction were purchased from Invitrogen, Life Technologies (Gaithersburg, MD). Reagents for real-time RT-PCR were obtained from Applied Biosystems (Foster City, CA).

### Cell culture of macrophages

SF was obtained from 14 patients with active RA, who were visiting our outpatient clinic. Local research ethics committee gave approval for the study and all patients had given informed consent. SF was diluted 1:1 with RPMI plus 10 mg/ml gentamicin. Subsequently mononuclear cells (MC) were isolated by Lymphoprep density gradient centrifugation. SFMCs were cultured (5 × 10^6 ^cells/ml) in 2 ml RPMI + 2% human pooled serum in 6-well plates (Costar, Badhoevedorp, the Netherlands) or in 1 ml in 12-well plates at 37°C in a 5% CO_2 _atmosphere. The cells that adhered after two hours were used for experiments. For hypoxia experiments cells were incubated in an hypoxia incubator, the Ruskinn Invivo_2 _200, with an O_2 _level of 1%.

THP-1 monocytic cells (ATCC-LGC, Middlesex, UK) were cultured in RPMI plus additives (25 mM HEPES, 200 nM glutamine, 100 mM Na-pyruvate, 10 mg/ml gentamicin, 0.05 M β-mercaptoethanol, 2.2 μg/ml amphotericin B) supplemented with 10% FCS and were differentiated into macrophages with 100 nM PMA (phorbol 12-myristate 13-acetate) during 3 days in RPMI plus 10% FCS and additives. Culture or stimulation periods are indicated where relevant.

### HIF-1α expression in rheumatoid synovial tissue and in THP-1 macrophages

Synovial tissue was obtained from RA patients (n = 8), who underwent synovectomy or joint replacement surgery, and who had given informed consent. Synovial tissue was formalin fixed and paraffin embedded, and 4 μM slides were cut. Sections were deparaffinised with xylene and rehydrated with ethanol and water. Endogenous peroxidase activity was blocked with 0.3% hydrogen peroxide in PBS. The sections were incubated overnight at 4°C with monoclonal antibody HIF-1alpha67sup. For detection, the sections were incubated with peroxidase labeled anti-mouse polymer from EnVision Kit (K4006, DAKO, Glostrup, Denmark). Sections were also stained for macrophages (CD68, clone PGM-1, DAKO), and vessels (CD31, clone JC70A, DAKO).

HIF-1α expression was detected by Western blotting in THP-1 macrophages stimulated with 1 μg/ml LPS for 6 hours or left unstimulated. Nuclear extracts were prepared with the NE-PER^® ^Nuclear and Cytoplasmic Extraction Reagents according to the manufacturers' instructions. Samples were loaded onto a 10% SDS-PAGE gel and resolved by running at 120 V and 15 Watt constant. Semidry blotting onto nitrocellulose membrane was followed by immunodetection with anti-HIF-1α (BD Pharmingen) and anti-mouse-immunoglobulins labeled with HRPO. Enhanced chemiluminescence (ECL) detection was performed according to the manufacturer's guidelines (Lumi-Light^plus^, Roche Diagnostics).

### mRNA expression of HIF-1α and VEGF

THP-1 cells (1 × 10^6^/ml) were cultured in 6-well plates and stimulated with 1 μg/ml LPS at different time points during differentiation. After 4 hours of stimulation total RNA was isolated from the cells with TRIzol reagent according to the manufacturers' instructions as described earlier [20]. DNAse treatment (Ambion, Huntingdon, Cambridgeshire, UK) was performed and subsequently cDNA was synthesized from 2.0 μg of total RNA using M-MLV Reverse Transcriptase and oligo (dT)_14-18_. For measurement of mRNA for HIF-1α, VEGF, IL-8, matrix metalloproteinase (MMP)-9 and glyceraldehyde-3-phosphate dehydrogenase (GAPDH) 1 μl of cDNA in triplicate was used for amplification by the Taqman real-time PCR system (ABI Prism 7900HT Sequence Detection System, Applied Biosystems, Foster City, CA) with specific Taqman primers/probes (Applied Biosystems). Amplification was performed using standard conditions and calculations of fold induction were performed as described earlier. The amount of target, normalized to an endogenous reference (GAPDH) and relative to the unstimulated control sample, is given by: 2^-ΔΔCT^. mRNA expression in SFM was determined in the same way.

### Determination of VEGF, IL-8, and MMP-9 levels in cell culture supernatants

Production of pro-angiogenic factors was measured in cell culture supernatants of THP-1 cells (0.5 × 10^6^/0.5 ml in 24 well plates) during differentiation either unstimulated or stimulated for 48 hours with 1 μg/ml LPS. Effects of YC-1, a specific HIF-1α inhibitor, and of kinase inhibitors (30 minutes pre-treatment) on protein production was also measured in macrophage cell supernatants after 48 hours LPS stimulation.

VEGF, IL-8, and MMP-9 levels were measured in cell supernatants by ELISA, using matched antibody pairs for ELISA and recombinant proteins as standards (R&D Systems). For optimal determination of MMP-9, precoating with F(ab)_2 _fragments of goat-anti-mouse IgG-Fc (Jackson, West Grove, Pennsylvania, USA) in 0.1 M carbonate buffer (pH = 9.6) for at least 48 hours was done before coating of the capturing antibody. In all ELISAs, after sample incubation and binding of the biotinylated detecting antibodies, color reaction was performed with streptavidin-poly-HRP (Sanquin, Amsterdam, the Netherlands) and tetramethyl-benzidin (TMB, Roth, Karlsruhe, Germany).

### Statistics

One-way ANOVA with Dunnett's post test was performed using GraphPad Prism version 4.00 for Windows, GraphPad Software (San Diego, California, USA).

## Results

### HIF-1α expression in rheumatoid synovial tissue

**Figure 1:**
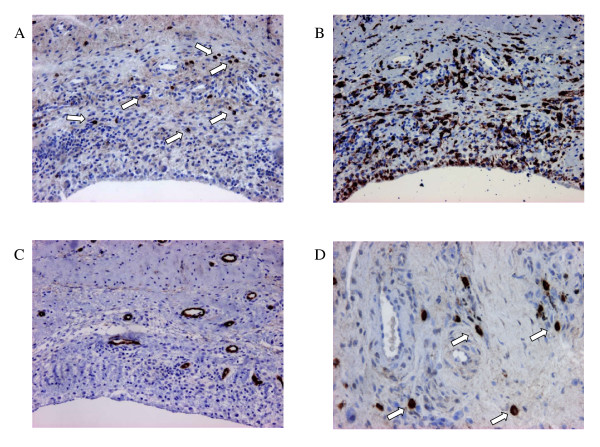
**Expression of HIF-1α, CD68, and CD31 in RA synovial tissue**. Representative pictures of immunohistochemical staining of RA synovial tissue obtained after total joint replacement. Consecutive (A, B, C) sections were treated according to standard procedures for immuno-histochemistry, and stained for HIF-1α (A, D), macrophage marker CD68 (B), and endothelial cell marker CD31 (C). Magnification A-C 200×, D 400×.

First we investigated expression of HIF-1α in RA synovial tissue. Following the staining procedure described by Zhong and Semenza [21] and using monoclonal antibody HIF-1alpha67sup we detected a nuclear staining of HIF-1α in synovial tissues from all RA patients, which was not restricted to the lining layer but had a diffuse pattern throughout the tissue (figure 1A and 1D, see arrows) [22]. Staining of synovial tissue of OA patients showed significantly less HIF-1α staining (data not shown). The synovial tissues also showed abundant staining for macrophages (CD68) (figure 1B) and vessels (CD31) (figure 1C).

### mRNA expression of HIF-1α and VEGF in THP-1 cells and synovial macrophages

**Figure 2:**
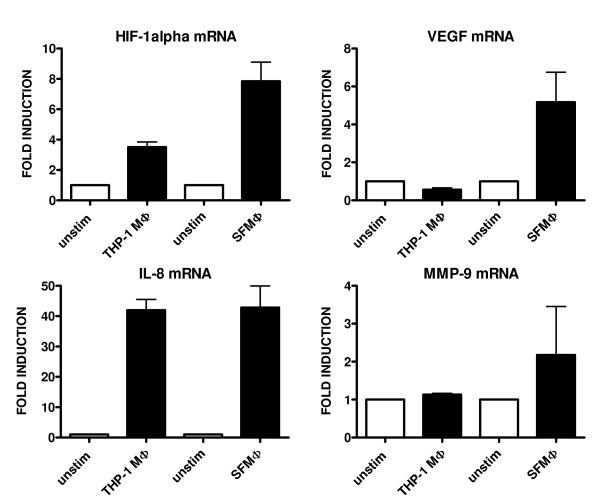
**mRNA expression of HIF-1α, VEGF, IL-8, and MMP-9 in macrophages under normoxia**. HIF-1α, VEGF, IL-8 and MMP-9 mRNA levels under normoxia from differentiated THP-1 cells (macrophages) and macrophages isolated from RA synovial fluid with LPS stimulation (4 hours) and without LPS stimulation. mRNA expression was expressed as fold induction (2^-ΔΔCt^, Ct is threshold value), which is normalized to a household gene (GAPDH) and relative to an unstimulated sample (fold induction = 1).

**Figure 3:**
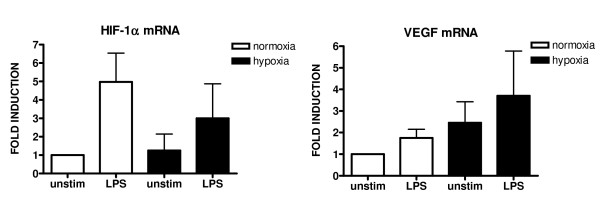
**mRNA expression of HIF-1α and VEGF in RA SF macrophages under normoxia and hypoxia**. HIF-1α and VEGF mRNA levels of synovial fluid macrophages (n = 4) under normoxic or hypoxic (1% O_2_) conditions with and without LPS stimulation. mRNA expression was expressed as fold induction (2^-ΔΔCt^, Ct is threshold value), which is normalized to a household gene (GAPDH) and relative to an unstimulated sample (fold induction = 1).

To investigate both mRNA and protein expression of HIF-1α in vitro we first measured levels of HIF-1α and VEGF mRNA in differentiated THP-1 cells and in macrophages from SF (n = 4) with realtime RTPCR. In figure 2 it is shown that HIF-1α mRNA expression is increased in THP-1 cells, and that macrophages isolated from RA SF have very high HIF-1α expression (mean fold induction of 7.9). VEGF mRNA levels were also increased in SF macrophages (mean fold induction of 5.2). IL-8 mRNA levels were increased 40-50 fold in both THP-1 and SF macrophages, and MMP-9 mRNA levels were two-fold higher in SF macrophages. Incubation of SF macrophages in an hypoxia incubator did not increase HIF-1α expression further, but did raise VEGF mRNA levels slightly (figure 3).

### HIF-1α protein expression is induced during differentiation under non-hypoxic conditions and can be blocked by kinase inhibitors

**Figure 4:**
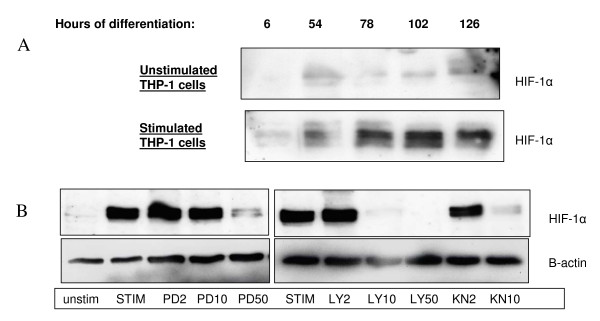
**HIF-1α protein expression in THP-1 monocytes and macrophages**. Protein expression of HIF-1α measured by Western Blotting in nuclear extracts of THP-1 cells. (A) HIF-1α expression during differentiation of THP-1 monocytes to macrophages in unstimulated and LPS-stimulated cells (6 hours). (B) Effect of different concentrations (2,10 or 50 μM) of kinase inhibitors on HIF-1α expression in LPS-stimulated THP-1 macrophages (cells were differentiated for 72 hours with 100 nM PMA): PD = PD98059-MEK inhibitor, LY = LY294002-PI3K inhibitor, KN = KN93-CAMKII inhibitor.

Next we continued with protein expression of HIF-1α during differentiation and after stimulation. THP-1 cells under non-hypoxic conditions were induced to differentiation with 100 nM PMA and HIF-1α expression was studied in LPS stimulated or unstimulated cells at several time points (figure 4A). We observed an increased HIF-1α expression during differentiation in unstimulated cells, which was even higher after LPS stimulation.

Then we investigated the effects of the specific MEK inhibitor PD98059, the PI3K inhibitor LY294002, and the CAMKII inhibitor KN93 on HIF-1α protein expression in differentiated THP-1 cells. Figure 4B shows that the MEK inhibitor PD has an inhibitory effect at 50 μM on HIF-1α expression in differentiated THP-1 cells, the PI3K inhibitor LY at 10 and 50 μM, and the CaMKII inhibitor KN at 10 μM. So these various signal transduction pathways are involved in LPS-induced HIF-1α expression in macrophages.

### Production of proangiogenic factors during differentiation of THP-1 cells

**Figure 5:**
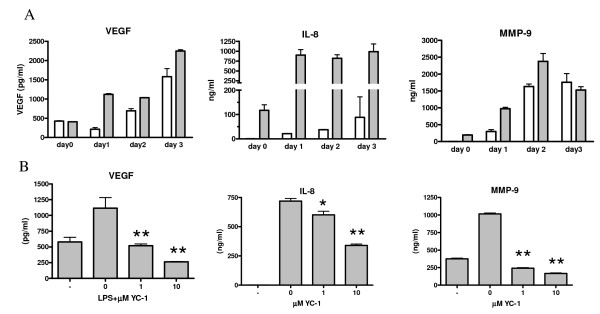
**Protein (VEGF, IL-8, and MMP-9) production of THP-1 cells during differentiation**. (A). THP-1 monocytic cells (n = 3) were differentiated for 3 days and were stimulated with LPS for 48 hours (hatched bars) or left unstimulated (open bars). Protein production of VEGF, IL-8 and MMP-9 was measured in cell supernatants by ELISA. In (B) the HIF-1α specific inhibitor YC-1 was added to the cultures (n = 3) and significant inhibition was seen for all proteins. Significance of inhibition was calculated compared to LPS stimulated sample (no inhibitor) with one-way ANOVA with Dunnett's post test: * p < 0.05, ** p < 0.01.

To see whether differentiation of THP-1 cells leads to increased production of pro-angiogenic factors, VEGF, IL-8 and MMP-9, protein levels were measured in cell supernatants of stimulated and unstimulated cells after 0, 1, 2 and 3 days of differentiation. As can be seen in figure 5A protein production of VEGF, MMP-9 and IL-8 increased during differentiation. Preincubation with the specific HIF-1α blocker YC-1 significantly inhibited VEGF-, IL-8- and MMP-9 production in THP-1 macrophages (figure 5B). From these results we can conclude that production of these angiogenic factors in macrophages is regulated by activation of HIF-1α.

### Regulation of VEGF, IL-8 and MMP-9 production

**Figure 6:**
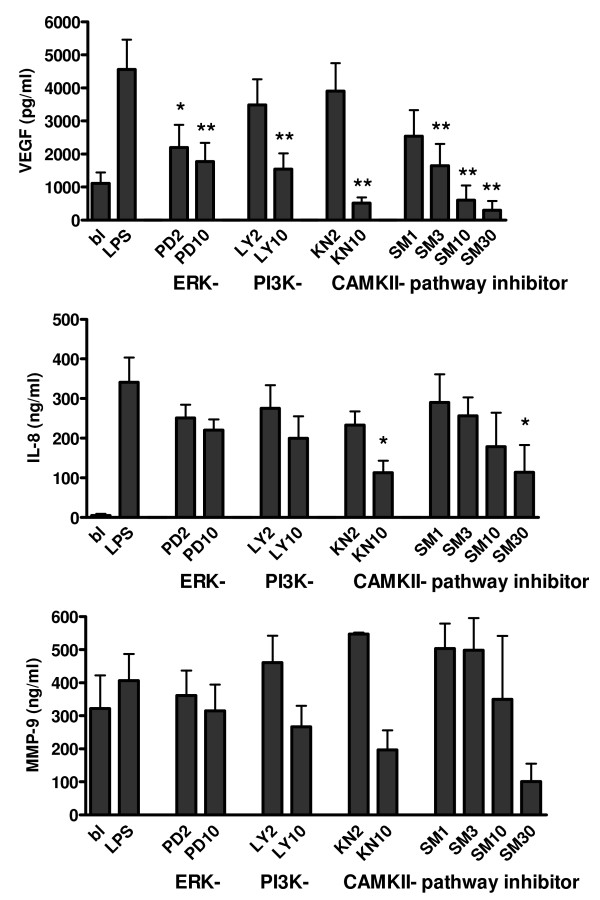
**Kinase inhibitor effects on VEGF, IL-8, and MMP-9 production by THP1 macrophages (n = 4)**. Differentiated THP-1 cells were stimulated for 48 hours with LPS and pretreated with different concentrations of inhibitors of ERK, PI3K, and CAMKII pathways. PD = PD98059-MEK inhibitor, LY = LY294002-PI3K inhibitor, KN = KN93-CaMKII inhibitor, SM = SMP114-CaMKII inhibitor. Significance of inhibition was calculated compared to LPS stimulated sample with one-way ANOVA with Dunnett's post test: * p < 0.05, ** p < 0.01.

To determine which intracellular pathways are involved in production of these angiogenic factors THP-1 cells were incubated with specific inhibitors of the ERK-, PI3K-, and CaMKII pathways. Since we had found effects of the CaMKII inhibitor KN-93 on HIF-1α expression we decided to include the novel CaMKII inhibitor SMP-114 (in phase 2 clinical trial for treatment of RA). Significant inhibition of VEGF production was seen with 10 μM PD, LY and KN, but also with 3 and 10 μM SMP-114 (figure 6). KN-93 at concentration 2 μM did not inhibit VEGF production in contrast to SMP-114 at 3 μM. From previous unpublished research we know that SMP-114 can also be used at higher concentrations (30 μM) than KN-93 without becoming cytotoxic. IL-8 production was significantly inhibited by CaMKII inhibitors (KN-93 at 10 μM and SMP-114 at 30 μM). MMP-9 production was slightly increased by LPS stimulation, but decreased by PI3kinase and CaMKII inhibitors (not significant).

**Figure 7:**
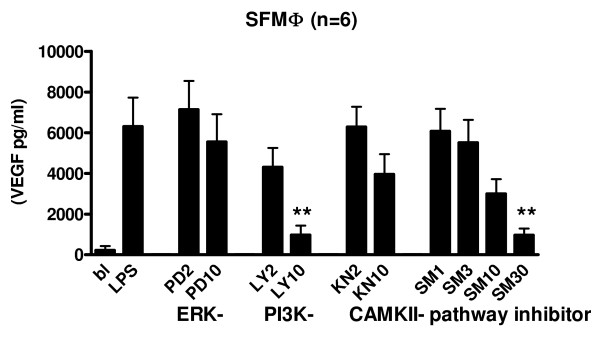
**Protein production in cell supernatants of RA SF macrophages**. Cells were stimulated with LPS and pretreated with different concentrations of inhibitors of ERK, PI3K, and CAMKII pathways. Protein production was measured in cell supernatants by ELISA. (A) VEGF production by SF macrophages (SFM) (n = 6). PD = PD98059-MEK inhibitor, LY = LY294002-PI3K inhibitor, KN = KN93-CaMKII inhibitor, SM = SMP114-CaMKII inhibitor. Significance of inhibition was calculated compared to LPS stimulated sample with one-way ANOVA with Dunnett's post test: * p < 0.05, ** p < 0.01.

We then performed these studies in SF macrophages. Figure 7 shows that VEGF production in SF macrophages was significantly reduced by the PI3K-inhibitor and the CaMKII inhibitor SMP-114. SMP-114 can be safely used at this concentration, whereas KN-93 can not. IL-8 production was not affected by signal transduction inhibitors (data not shown). As stimulation of SF macrophages with LPS reduced the high constitutive production of MMP-9, inhibitors were also added to unstimulated cells. MMP-9 production was inhibited by PI3K and CaMKII inhibitors, but this did not reach statistical significance (data not shown).

**Figure 8:**
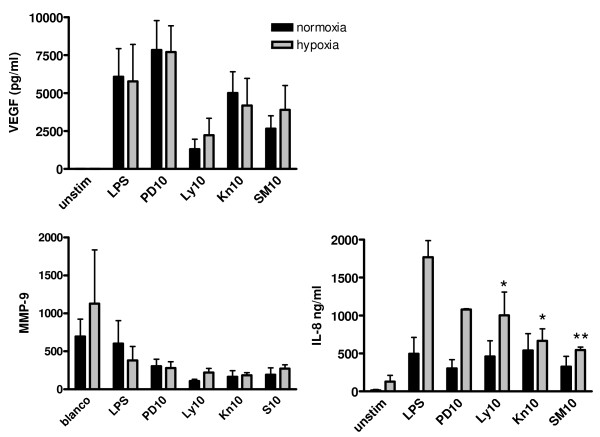
**VEGF, IL-8 and MMP-9 production measured in LPS-stimulated cell supernatants of RA SF macrophages under normoxia and hypoxia**. Cells were stimulated with LPS for 48 hours and pretreated with inhibitors (10 μM) of ERK, PI3K, and CAMKII pathways in a normal incubator (black bars) or in a hypoxia incubator (1% O_2_, Striped bars). PD = PD98059-MEK inhibitor, LY = LY294002-PI3K inhibitor, KN = KN93-CaMKII inhibitor, SM = SMP114-CaMKII inhibitor. Significance of inhibition was calculated compared to LPS stimulated sample with one-way ANOVA with Dunnett's post test: * p < 0.05, ** p < 0.01.

Since we detected an increase in VEGF mRNA expression in SF macrophages that were incubated in an hypoxia incubator, protein production was also measured under these circumstances. Figure 8 shows that VEGF and MMP-9 production did not increase when macrophages were stimulated with LPS in an hypoxia incubator compared to a normoxic incubator. However, PI3K and CaMKII inhibitors reduced VEGF levels as was seen under normoxia. Levels of IL-8 highly increased when the cells were incubated in hypoxia, and significant reduction was achieved with PI3K- and CaMKII inhibitors.

## Discussion

In this study we showed that HIF-1α is expressed in synovial tissue from rheumatoid arthritis patients, and also in macrophages isolated from RA SF. In the inflammatory, non-hypoxic regulation of HIF-1α expression both PI3kinase and CaMKII pathways are involved, which is reflected by significant reduction in VEGF levels by specific inhibitors.

Expression of HIF-1α, the inducible part of the transcription factor HIF-1, has been described for RA synovial tissue [23,24] especially in macrophages in the synovium [25]. However contradicting results have been reported demonstrating either nuclear or cytoplasmic staining, and with or without differences between RA and OA synovial tissue [23-25]. In the field of oncology, in which many publications report HIF-1α staining, the procedure as described by Semenza's group is considered the standard staining [21]. They described in different tissues a nuclear staining of HIF-1α, mostly with a diffuse pattern or located near necrotic areas or neovascular areas. We followed these staining procedures and found nuclear staining in 8 synovial specimens, both in the lining and in the sublining layer. Although we did not perform double staining it is likely that HIF-1α was expressed mainly by macrophages since these cells are found everywhere in the tissue. In contrast to one study [23] but in accordance with others [25], we found minor HIF-1α expression in OA synovial tissue [22]. This is in line with the nature of the tissue being inflammatory and angiogenic in RA, and less inflammatory in osteoarthritis synovial tissue.

Stabilization of HIF-1α can take place under hypoxic conditions but can also be induced by differentiation of monocytes to macrophages and by stimulation with LPS [11,12]. Macrophages isolated from RA SF come from an hypoxic environment [3], which was reflected by their high HIF-1α and VEGF mRNA levels compared to macrophages derived from THP-1 cells. Incubating these cells in an hypoxia incubator did not increase HIF-1α expression further since these cells already were hypoxic. By Western blotting we demonstrated that HIF-1α protein expression can be inhibited by the PI3kinase inhibitor and the CaMKII inhibitor KN93 at 10 μM in THP-1 macrophages, so there is a role for CaMKII signalling in HIF-1 regulation.

Induction of HIF-1α expression leads to production of angiogenic proteins. Both VEGF and MMP-9 levels increased during differentiation without stimulation with LPS, and this was further increased following stimulation. IL-8 production was also induced but highly increased after stimulation with LPS. When we used YC-1, (3-(5'-hydroxymethyl-2'-furyl)-1-benzyl indazole), which is considered a specific HIF-1α inhibitor) [26,27], levels of VEGF and MMP-9 were completely reduced whereas IL-8 levels were less diminished. This implies that VEGF and MMP-9 production are under control of HIF-1, whereas this is partly the case for IL-8. It has been reported that YC-1 can induce apoptosis *in vitro *in cell-lines, but this is primarily at concentration higher than 5 μM, so the reduction that was seen at 1 μM is due to blocking of HIF-1 activity [28].

Incubating THP-1 macrophages with different concentrations of the signal transduction inhibitors gave a significant reduction of VEGF protein levels at 10 μM or lower concentrations for all inhibitors, but for SF macrophages this was only the case for the PI3kinase inhibitor and for SMP-114. There is often a difference between cell lines and primary cell cultures, but the data convincingly show that these pathways are crucial in HIF-1 induced VEGF production. Incubation of SF macrophages in an hypoxia incubator did not increase VEGF or MMP-9 protein production, while IL-8 production was increased. Apparently hypoxia and LPS work synergistically in induction of IL-8, which still can be inhibited by PI3K- and CaMKII inhibitors. MMP-9 levels were decreased in SFM after stimulation with LPS. Lee *et al. *[29] showed that in serum of conditioned media inhibitory factors are present that inhibit MMP-9 production by macrophages. Since we cultured SFM in RPMI supplemented with 2% human pooled serum, it could well be that this is the reason for suppression of MMP-9 production.

Recently it was reported that in the mouse macrophage cell line RAW264.7 LPS induced activation was enhanced by hypoxia, resulting in increased TNF-α secretion [30]. Also, Fang *et al. *showed that HIF-1 and HIF-2 are important transcriptional effectors in primary macrophages experiencing hypoxia, more important than NF-κB [31]. In another recent publication it was shown that LPS induces intracellular calcium release in macrophages and that CaMKII is activated after LPS-induced TLR-activation [19]. It was demonstrated that CaMKII activation directly induces cytokine production in macrophages. From these studies is clear that both hypoxia and inflammation are important in macrophage activation and that different signal transduction pathways are involved.

In this study we confirm the involvement of the PI3kinase pathway in HIF-1α regulation in THP-1 macrophages and macrophages from RA SF. We suspected a role for CaMKII inhibition initially based on a report by Yuan *et al*., in which they mentioned that HIF-1 transcriptional activity was dependent on CaMKII activation [17]. In our study we found that CaMKII inhibition reduces HIF-1α expression and VEGF production in stimulated macrophages. In inflammatory conditions such as RA the relevance of HIF-1 primarily lies in controlling angiogenesis, since this is an important feature of RA. Inhibition of angiogenesis has already been investigated in a number of animal arthritis studies, via drug intervention [15], or by gene therapy [32] in rat models of arthritis. In the introduction we already mentioned animal studies with specific HIF-1 inhibitors. In humans anti-angiogenic effects are known for some drugs, for instance anti-TNF therapy induced reduction of VEGF levels in RA patients [33]. Anti-angiogenic effects are in our study now established for the CaMKII inhibitor SMP-114 in macrophages. However, this is clearly an off-target effect and although beneficial in this case effects like these need further investigation in new developed drugs.

## Conclusions

In this study we demonstrated inhibition of HIF-1α protein expression and significant inhibition of VEGF production by CaMKII inhibitors. This is an unknown but very interesting effect of the CaMKII inhibitor SMP-114, which is now in clinical trial as DMARD for the treatment of rheumatoid arthritis. This effect may contribute to the anti-arthritic effects of SMP-114.

## Competing interests

The authors have received an unrestricted grant from Dainippon Sumitomo Pharma for their research in general. The SMP-114 was provides by Dainippon Sumitomo Pharma free of charge. Dainippon Sumitomo Pharma was not involved in the planning and execution of this study. The authors have no financial interests whatsoever in this company and therefore declare that they have no competing interests.

## Authors' contributions

JW and EB conceived and designed the study, IAMvR and BDvdM performed the experiments. MAvL, and MDP participated in interpretation of data. JW, EB, and CGMK have been involved in writing the manuscript. All authors read and approved of the final manuscript.

## Pre-publication history

The pre-publication history for this paper can be accessed here:

http://www.biomedcentral.com/1471-2474/11/61/prepub
